# Supplementary material for: Rapid turnover of life-cycle-related genes in the brown algae
Source: Genome Biol. 2019 Feb 14;20:35. doi: 10.1186/s13059-019-1630-6 (PMC6374913; doi:10.1186/s13059-019-1630-6)
Supplement: Supplementary file 3 — Protein alignments, corresponding Gblocks html file and CDS sequences for the lineage conserved gametophyte-biased genes. (ZIP 2109 kb) [file 13059_2019_1630_MOESM3_ESM.zip › Con_GA_alignments/Ec-02_005530.aa_ali.fasta-gb.htm]

Ec-02\_005530.aa.fasta.trans.aa\_ali.fasta


## Gblocks 0.91b Results

Processed file: **Ec-02\_005530.aa.fasta.trans.aa\_ali.fasta**  
Number of sequences: **2**  
Alignment assumed to be: **Protein**  
New number of positions: **130** (selected positions are underlined in blue)

```
                         10        20        30        40        50        60
                 =========+=========+=========+=========+=========+=========+
Ec-02_005530     --------------------------------------------------MHSVIRIARR
MSTRG.14888.1    GVEEEKNDDEEEVLAGCVEEFYEEQGVETLVSAMEHHVHDASLLLAAWQAMHSVIRVARR
                                                                   ##########


                         70        80        90       100       110       120
                 =========+=========+=========+=========+=========+=========+
Ec-02_005530     EQSPKDPPAGHVTK---DGP----------ERLSDNRVSEALHR-GNKLDQQASLLAQVM
MSTRG.14888.1    TLPKTSTAHNAATKSDGDGTSLDIVLARAGAPLPRRDLSEPLLRRGKNLEELLSLLARTM
                                                 ############ ###############


                        130       140       150       160       170       180
                 =========+=========+=========+=========+=========+=========+
Ec-02_005530     DVHQAVAPLLWICLVCLIDLHALEARVSDIFIINAGCQVLHRCIRINARHTAVVEQVCRV
MSTRG.14888.1    DAHEAVAPLQWICLVCLIDLHQLEARVSDIFMLRSGCQILNRCIRTNARHTAVVEQGCRA
                 ############################################################


                        190       200       210       220       230       240
                 =========+=========+=========+=========+=========+=========+
Ec-02_005530     VAVMSEPSFTSRTRFMVENIQDAIHEHKLVGDRTN--YVLG-RVLKLLP--------LPP
MSTRG.14888.1    VAVMSEPSFTSRTRLFVEKVQDVIHEVVFLHKRHPGVNKLGNRALDALWGKDDFGAKQKA
                 #################################                           


                        250
                 =========+======
Ec-02_005530     PPP------------Q
MSTRG.14888.1    SAAERFHTKADEEGDE
```

```
Parameters used
Minimum Number Of Sequences For A Conserved Position: 2
Minimum Number Of Sequences For A Flanking Position: 2
Maximum Number Of Contiguous Nonconserved Positions: 8
Minimum Length Of A Block: 10
Allowed Gap Positions: None
Use Similarity Matrices: Yes
```

```
Flank positions of the 3 selected block(s)
Flanks: [51  60]  [93  104]  [106  213]  

New number of positions in Ec-02_005530.aa.fasta.trans.aa_ali.fasta-gb:  130  (50% of the original 256 positions)
```
